# Canine Distemper Virus Alters Defense Responses in an Ex Vivo Model of Pulmonary Infection

**DOI:** 10.3390/v15040834

**Published:** 2023-03-24

**Authors:** Elisa Chludzinski, Małgorzata Ciurkiewicz, Melanie Stoff, Johanna Klemens, Johannes Krüger, Dai-Lun Shin, Georg Herrler, Andreas Beineke

**Affiliations:** 1 Department of Pathology, University of Veterinary Medicine Hannover, 30559 Hannover, Germany; 2 Center for Systems Neuroscience (ZSN), 30559 Hannover, Germany; 3 Institute of Virology, University of Veterinary Medicine Hannover, 30559 Hannover, Germany; 4 Department of Veterinary Medicine, College of Veterinary Medicine, National Chung Hsing University, Taichung 402, Taiwan

**Keywords:** canine distemper virus, ciliary activity, cytokines, immunohistochemistry, morbillivirus, MHC-II, precision-cut lung slices, viral pneumonia

## Abstract

Canine distemper virus (CDV), belonging to the genus *Morbillivirus*, is a highly contagious pathogen. It is infectious in a wide range of host species, including domestic and wildlife carnivores, and causes severe systemic disease with involvement of the respiratory tract. In the present study, canine precision-cut lung slices (PCLSs) were infected with CDV (strain R252) to investigate temporospatial viral loads, cell tropism, ciliary activity, and local immune responses during early infection ex vivo. Progressive viral replication was observed during the infection period in histiocytic and, to a lesser extent, epithelial cells. CDV-infected cells were predominantly located within the bronchial subepithelial tissue. Ciliary activity was reduced in CDV-infected PCLSs, while viability remained unchanged when compared to controls. MHC-II expression was increased in the bronchial epithelium on day three postinfection. Elevated levels of anti-inflammatory cytokines (interleukin-10 and transforming growth factor-β) were observed in CDV-infected PCLSs on day one postinfection. In conclusion, the present study demonstrates that PCLSs are permissive for CDV. The model reveals an impaired ciliary function and an anti-inflammatory cytokine response, potentially fostering viral replication in the lung during the early phase of canine distemper.

## 1. Introduction

Viral infections of the lower respiratory tract are a major cause of disease and mortality. Morbilliviruses, belonging to the family *Paramyxoviridae*, comprise highly contagious pathogens and cause regular outbreaks in humans and animals worldwide [1]. Members include canine distemper virus (CDV), measles virus (MeV), rinderpest virus (eradicated since 2011), peste des petits ruminants virus, phocine distemper virus, feline morbillivirus, and cetacean morbillivirus (CeMV) [2]. A hallmark of morbillivirus infections is generalized immunosuppression due to leukopenia, and impaired adaptive immunity, favoring opportunistic infections [3,4]. CDV possesses a broad host range, and its high fatality rate in unprotected populations represents a threat for many endangered wildlife species [5]. Due to its neuro-, lympho-, and epitheliotropism, CDV induces a systemic disease, including demyelinating leukoencephalitis, catarrhal enteritis, and interstitial pneumonia [6]. Following inhalation, the virus is encountered by immune cells, such as alveolar macrophages and dendritic cells, reaching the airway surface and expressing the viral receptor signaling lymphocyte activation molecule (SLAM). Subsequently, the virus is transported through the respiratory epithelium to local lymphoid tissues [7,8,9]. After viral replication in lymphocytes and histiocytes, the virus disseminates by cell-associated viremia and re-enters the lung hematogenously [10]. Here, infected immune cells migrate to the basolateral side of epithelial cells, which are infected by CDV by the adherence protein nectin-4, representing the second virus entry receptor [11,12,13].

As airborne transmission plays a central role for morbillivirus spread, respiratory epithelial cells, as part of the innate immune system, provide a first mechanical and chemical barrier against viral infection. Airway epithelial cells secrete mucus and propel pathogens towards the outer environment by coordinated ciliary beating (mucociliary clearance) [14]. Other innate immune mechanisms include detection of viral structures by pattern recognition receptors, and presentation of viral antigens by the surface protein major histocompatibility complex class II (MHC-II) to CD4^+^ T cells to promote virus-specific adaptive immune responses [15]. In the lung, besides professional antigen-presenting cells (dendritic cells/macrophages), pneumocytes and respiratory epithelial cells are able to express MHC-II under inflammatory conditions [16]. The interplay between different cells involved in pulmonary immune responses is orchestrated by cytokines [17]. Here, proinflammatory cytokines, such as tumor necrosis factor alpha (TNF-α), IL-1, IL-6, and IL-12, are involved in virus clearance and progression of inflammatory lesions, with macrophage polarization towards a proinflammatory M1 type. By contrast, cytokines such as IL-10 and TGF-β exhibit inhibitory effects on host immune responses and may facilitate viral persistence, evoking an M2-type macrophage response [18,19,20].

Traditionally, morbillivirus pathogenesis has been studied experimentally in in vivo models, using nonhuman primates and cotton rats for MeV infection, and ferrets and dogs for CDV infection [21,22,23,24]. However, high costs of maintenance infrastructure of laboratory animals, low availability of tissue of diseased animals and humans, and decreased public acceptance of animal experiments raised efforts to implement alternative methods according to the 3R principles (refinement, reduction, replacement) in infection research [25,26]. Therefore, in vitro models have been established to study infectious diseases, including those addressing respiratory morbillivirus infection, e.g., MeV infection of primary differentiated respiratory epithelial cells, and CDV infection of air–liquid interface cultures [27,28]. However, these approaches consist of a limited range of cell types and show a metabolism adapted to culture conditions, and therefore do not optimally mimic the in vivo condition. Three-dimensional ex vivo models, such as precision-cut lung slices (PCLSs), have been established in several research fields, such as toxicology and infection studies, and may in part overcome these limitations [29,30]. PCLSs include several cell types and matrix elements, such as epithelial cells, connective tissue, immune cells and vascular structures in their organotypic anatomical architecture. Moreover, numerous PCLSs can be generated from one lung lobe, and uniformity in size and thickness can be arranged using precise tissue slicers [31]. In this respect, ex vivo organotypic models have the potential to complement and partially replace in vivo research.

These advantages of PCLSs highlight the huge potential of ex vivo tissue explant cultures in infection research, besides their contributory role to animal welfare. Currently, only few data exist regarding ex vivo CDV infection in the lung. To add further work to this promising field of CDV research, the present study provides the first characterization of CDV-infected canine PCLSs [32,33,34]. Accordingly, the present research aimed at utilizing a canine PCLS model of CDV infection to describe viral loads, cell tropism, ciliary activity, MHC-II expression, and cytokine responses to test the hypothesis that CDV infection interferes with pulmonary defense mechanisms during the initial phase of canine distemper.

## 2. Materials and Methods

### 2.1. Lung Tissue

For preparation of PCLSs, lung tissue was removed from dogs submitted to routine necropsy service within 1–4 h post mortem (in total 118 slices derived from 6 dogs). All dogs were vaccinated against CDV. The animals were euthanized due to poor clinical prognosis (Supplementary Table S1). Sample selection was based on the absence of pathological lesions and history of clinical lung disease. No animals were killed for the purpose of this study and written consent was given by the owners.

### 2.2. Virus

For PCLS infection, the CDV strain R252, originally isolated from a spleen homogenate of a naturally CDV-infected dog (kindly provided by Prof. S. Krakowka, Ohio State University, Columbus, OH, USA) was used [35]. The virus was propagated in Vero cells (passage 26) with a titer of TCID_50_ 10^5.25^/100 µL.

### 2.3. Preparation of Precision-Cut Lung Slices

**Figure 1:**
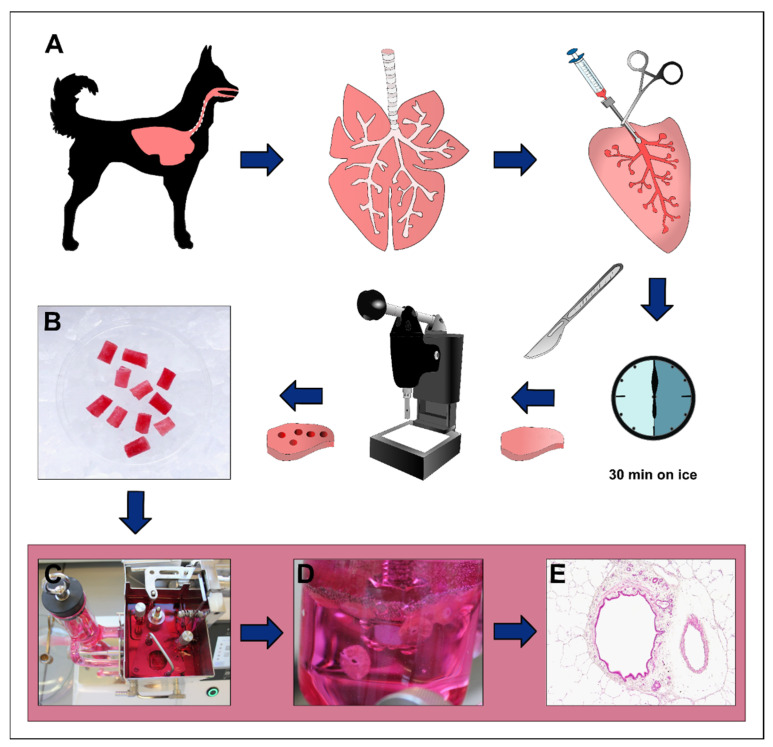
Generation of precision-cut lung slice (PCLS). Lung tissues are removed from dogs, followed by inflation of 1.5% low-melting agarose through the airways of single lung lobes and solidification on ice for 30 min (**A**). Lung lobes are further cut into 1 cm thick slices and cylindric pieces containing a cross-sectioned bronchus are generated (**B**). These are inserted into a tissue slicer (**C**), which produces a uniform PCLS of 250 µm thickness (**D**). Hematoxylin-eosin (HE)-stained PCLS section shows retained pulmonary tissue architecture (**E**).

Preparation of organotypic PCLSs was performed as described previously with slight modifications [36]. The workflow is schematized in Figure 1. Briefly, the entire pluck was resected from the carcass with sterile cutlery and kept on ice until further processing. To reduce tissue damage during cutting, inflation of the lung through the airways with a 1.5% low-melting agarose solution was required. This solution was prepared by dissolving 6 g of low-melting agarose (Gerbu Biotechnik GmbH, Heidelberg, Germany) in 200 mL sterile distilled water with a stirring magnet, and subsequent heating in a microwave until complete melting of the agarose. Roswell Park Memorial Institute (RPMI) medium was generated using Gibco^TM^ RPMI 1640 medium powder according to the manufacturer’s protocol (Gibco, Life Technologies, Paisley, UK). Prior to effusion, 200 mL of sterile double-concentrated RPMI medium were added to the solution and further stirred with a sterile magnet at 37 °C. Lung lobes were pinched off with a squeeze, removed from the pluck, and sterile gavage needles were inserted through the large bronchi. The agarose medium solution was slowly injected into the airways by a sterile syringe and the gavage needles, followed by solidification of the inflated lung lobe on ice for 30 min. The lung lobe was cut manually in approximately 1 cm thick slices with a scalpel. Subsequently, cylindric tissue cores measuring 8 mm in diameter and containing a centrally located bronchus were punched out with a tissue coring press (Alabama Research and Development, MD5000, Munford, AL, USA) and cut into 250 µm thick slices in a tissue slicer (Alabama Research and Development, MD6000, Munford, AL, USA) filled with culture medium (single-concentrated RPMI 1640 (Gibco), supplemented with 1% penicillin–streptomycin (Sigma-Aldrich Chemie GmbH, Steinheim, Germany), and 100 µL enrofloxacin (Baytril^®^ 50 mg/mL, Elanco GmbH, Cuxhaven, Germany) at a rate of 25 slices/min. Slices were gently transferred to 6-well plates prefilled with culture medium (5 slices per well) and incubated at 37 °C in 5% CO_2_. To remove the agarose, PCLSs were gently washed 3 times by pipetting in a cell strainer (Fisherbrand™, 100 µm mesh size, Fisher Scientific GmbH, Schwerte, Germany) and the medium was changed every 30 min for 3 times. Subsequently, PCLSs were transferred into 24-well plates prefilled with 1 mL culture medium (1 slice per well) and incubated overnight at 37 °C at 5% CO_2_.

### 2.4. Evaluation of Ciliary Activity

To check the viability of generated PCLSs, ciliary beating activity was assessed by light microscopy (Olympus IX70-S8F2, Olympus Life Science Europe GmbH, Hamburg, Germany) at 24 h after isolation. The circumference of each bronchus was optically divided into ten parts of equal size to evaluate the presence or absence of ciliary movement as described (Supplementary Figure S1) [36,37]. An exemplary video showing intact ciliary beating is shown in Supplementary Video S3. Slices with 90–100% ciliary beating activity were chosen for further infection experiments. Following CDV infection, ciliary beating activity was assessed immediately after infection (0 days), and on 1, 3, and 6 days postinfection (dpi).

### 2.5. Infection of Precision-Cut Lung Slices with Canine Distemper Virus

PCLSs were infected with CDV at 24 h after isolation. Prior to infection, PCLSs were transferred to new 24-well plates and washed 3 times with phosphate-buffered saline (PBS, Sigma-Aldrich, Schnelldorf, Germany) supplemented with 1% penicillin–streptomycin (Sigma-Aldrich), followed by inoculation of 300 µL CDV-R252-culture medium suspension (4 °C) with an infection dose of 30,000 virus particles per well. Uninfected control slices were incubated with 300 µL culture medium (4 °C) instead of the virus-medium suspension. After 2 h of incubation at 37 °C and 5% CO_2_, slices were washed 3 times with PBS (Sigma-Aldrich, D8538) supplemented with 1% penicillin–streptomycin (Sigma-Aldrich, P4333), followed by addition of 1 mL fresh culture medium. Subsequently, slices were cultured for 1, 3, or 6 days until harvesting. For the 6 dpi group, the culture medium was replaced by fresh medium at 3 dpi. During sampling, slices were either immersion-fixed in 10% neutrally buffered formalin and embedded in paraffin the next day (72 slices derived from 6 dogs), snap-frozen with liquid nitrogen in O.C.T. embedding compound for molecular analyses (38 slices derived from 4 dogs), or immersion-fixed in glutaraldehyde for ultrastructural examination (8 slices derived from 3 dogs). Supernatants of each well were collected separately for lactate dehydrogenase (LDH) assay analysis. Formalin-fixed and paraffin-embedded (FFPE) lung slices were cut serially at 2 µm with a rotational microtome (Reichert-Jung BC2030, Leica Biosystems, Nussloch, Germany) and mounted on Superfrost^®^ Plus (Gerhard Menzel GmbH, Braunschweig, Germany). Subsequently, they were subjected either to hematoxylin-eosin (HE) staining for morphological evaluation, immunohistochemistry, in situ hybridization, or immunofluorescence.

### 2.6. Immunohistochemistry

To detect CDV antigen, immunohistochemical staining of PCLSs was performed using the EnVision^+^ detection system (Dako, Glostrup, Denmark). For MHC-II detection, the avidin–biotin–peroxidase complex method was used. FFPE slides (72 slices derived from 6 animals) were deparaffinized by ROTICLEAR^®^ (Carl Roth GmbH + Co. KG, Karlsruhe, Germany) with subsequent rehydration through a series of graded alcohols for 3 min each. Endogenous peroxidase activity was suppressed with H_2_O_2_ (0.5%) in 85% ethanol for 30 min, followed by heat-induced antigen retrieval by incubation of the samples in citrate buffer (pH 6.0) for 20 minutes in a microwave (800 W). To block unspecific reactions of the secondary antibody, slides were incubated with normal goat serum for 20 min (MHC-II only). The primary antibodies (listed in Table 1) were diluted in PBS with 1% bovine serum albumin (BSA; Carl Roth) and incubated overnight (18 h, 4 °C). For negative controls, the primary antibody was substituted with ascites fluid from nonimmunized BALB/c mice. Subsequently, slides used for MHC-II immunostaining were incubated with a polyclonal biotinylated secondary antibody (goat anti-mouse, Vector Laboratories, Burlingame, CA, USA, BA-9200) for 45 min (room temperature) with a subsequent 20 min treatment (room temperature) with the avidin–biotin–peroxidase complex (Vectastain Elite ABC Kit, Vector Laboratories). For immunostaining of CDV-N (nucleoprotein), slides were incubated with 100 µL of the EnVision^+^ peroxidase-labelled polymer as secondary antibody for 30 min (room temperature). The antigen–antibody reactions were visualized by incubation in PBS with 3,3′-diaminobenzidine tetrahydrochloride (0.05%; Merck KGaA, Darmstadt, Germany) and H_2_O_2_ (0.03%) for 5 minutes, followed by nuclear counterstaining (30 s) with Mayer’s hemalaun (Carl Roth).

Quantitative analysis was carried out manually using a light microscope (Carl Zeiss, Jena, Germany). Absolute numbers of CDV- and MHC-II-positive cells were counted separately within alveolar regions, subepithelial connective tissue of bronchi, and bronchial epithelia using a morphometric grid in at least four representative high-power fields (equaling 0.0625 mm^2^). The mean of positive cells per observed field was calculated and unified as a value of positive cells/mm^2^.

### 2.7. Immunofluorescence Double-Labelling

In order to study the cell tropism of CDV in PCLSs, immunofluorescence staining to detect CDV, ionized calcium-binding adapter molecule 1 (Iba-1, histiocytic cell marker), and cytokeratin (epithelial cell marker) were performed on formalin-fixed and paraffin-embedded PCLSs (8 slices derived from 3 dogs) as described [38]. Deparaffinization by ROTICLEAR^®^ (Carl Roth), rehydration through graded alcohols, and rinsing three times in PBS with a stirring tool (5 min each) were followed by incubation in citrate buffer (pH 6.0) in a microwave (800 W, 20 min). Unspecific secondary antibody reactions were blocked by incubation with 20% goat normal serum in PBS with 1% BSA and 0.1% Triton X-100 (Sigma-Aldrich, St. Louis, MO, USA) for 30 min. Primary antibodies (anti-CDV-N, either combined with anti-cytokeratin or anti-Iba-1, detailed in Table 1) were diluted together in PBS with 1% BSA and 0.1% Triton X-100 and incubated overnight (18 h, 4 °C). For negative controls, ascites fluid from nonimmunized BALB/c mice and rabbit normal serum were used instead of the primary antibodies. Secondary antibodies (goat anti-mouse Alexa Fluor 488 (Jackson ImmunoResearch, Newmarket, UK, 115-545-003, polyclonal) and goat anti-rabbit Cy3 (Jackson ImmunoResearch, 111-165-144, polyclonal) were diluted at 1:200 in PBS with 1% BSA and Triton X-100 with subsequent incubation for 45 minutes at room temperature in the dark, followed by rinsing in PBS for 3 × 5 min. Nuclei were counterstained with bisbenzimide (Sigma-Aldrich; 1:100 in sterile double-distilled water) for 5 min, and slides were mounted with fluorescence mounting medium (Dako, Glostrup, Denmark).

Slides were evaluated for the presence of colocalization by fluorescence microscopy (Keyence BZ-9000E with BZ-II-analyzer software BZ-H2AE, hard-coated filters: Cy3 (emission 624/40 nm, excitation 562/40 nm), AlexaFluor^®^488 (emission 520/35 nm, excitation 472.5/30 nm), bisbenzimide (emission 447/60 nm, excitation 377/55 nm), Keyence, Mechelen, Belgium).

### 2.8. In Situ Hybridization

To demonstrate CDV nucleoprotein mRNA, in situ hybridization using a dioxigenin (DIG)-labelled RNA probe was used as described previously [39,40,41]. In brief, FFPE PCLS sections (11 slices derived from 4 dogs) were deparaffinized with ROTI^®^Histol (Carl Roth), hydrated in a descending series of ethanol, and rinsed in ultrapure, double-distilled, diethyl pyrocarbonate (DEPC)-treated water. Subsequently, after predigestion with 0.2 M HCl for 20 min at room temperature and incubation in 2x standard saline citrate with 5 mM ethylenediaminetetraacetic acid at 50 °C for 30 min twice, proteolytic digestion was achieved with proteinase K (1 μg/mL, Roche Diagnostics, Mannheim, Germany) for 15 min at 37 °C, followed by postfixation with 4% paraformaldehyde. Acetylation was accomplished by 0.25 acetic anhydride in 0.1 M triethanolamine in double-distilled, DEPC-treated water for 30 min at room temperature. To stop unspecific bindings, prehybridization was performed for 60 min at 52 °C in a buffered RNA solution (250 µg/mL), with subsequent hybridization overnight in a moist chamber at 52 °C with a probe concentration of 100 ng/mL, using an RNA probe synthetized by a T3 RNA polymerase (Roche Diagnostics, Mannheim, Germany). The following DNA oligonucleotides were used for amplification of the gene sequences included in the template for the generation of the RNA probe (GenBank accession number: X02000; 5′-3′): forward primer (position 635–655): ACA GGA TTG CTG AGG ACC TAT; reverse primer (position 901–921): CAA GAT AAC CAT GTA CGG TGC [39]. The next day, following several post-hybridization washing steps, blocking of ribonucleases (30 min at 37 °C) and treatment with sterile sheep normal serum for 30 min at room temperature, an anti-DIG antibody conjugated with alkaline phosphatase (Roche Diagnostics, Mannheim, Germany, 1:200 in TRIS-NaCl-buffer, with 10% Triton-X and sheep normal serum, pH 7.5) was incubated for 2 h at room temperature. The reaction was visualized with the substrates nitroblue tetrazoliumchloride (Sigma-Aldrich Chemie, Taufkirchen, Germany) and 5-Bromo-4-chloro-3-indolyl phosphate (Sigma-Aldrich Chemie, Taufkirchen, Germany) in the dark for 16 h, yielding a purple precipitate. The slides were mounted with glycergel mounting medium (Dako, Glostrup, Denmark). CDV-infected DH82 cells served as positive control, and negative controls were treated with probe-free hybridization buffer.

### 2.9. Transmission Electron Microscopy

To assess ultrastructural morphology, glutaraldehyde-fixed PCLSs (8 slices derived from 3 dogs) were rinsed in 0.1 M cacodylate buffer (Serva Electrophoresis GmbH, Heidelberg, Germany) for 5 h on a roll mixer, followed by postfixation with 1% osmium tetroxide (Carl Roth GmbH + Co. KG, Karlsruhe, Germany). After dehydration through a series of graded alcohols, PCLSs were embedded in epoxy resin as described previously [42]. Selected representative sections were prepared as ultrathin sections, were contrasted with uranyl acetate and lead citrate, followed by evaluation by transmission electron microscopy (Carl Zeiss LEO EM 906, Leo Elektronenmikroskopie GmbH, Oberkochen, Germany).

### 2.10. Lactate Dehydrogenase (LDH) Assay

To monitor changes in the viability of PCLSs during the cultivation period, an LDH assay was performed. A duplicate of 50 µL of each supernatant was pipetted into a 96-well plate, as well as 50 µL of the working solution, prepared according to the manufacturer’s instructions (Cytotoxicity Detection Kit (LDH), Roche, Basel, Switzerland). After 20 min at room temperature and in the darkness, the absorbance was measured at 492 nm (reference: 630 nm) using a microplate reader (Fluostar Optima, BMG Labtech, Ortenberg, Germany). Both measurements were repeated three times, and the arithmetic mean of the reference measurement was subtracted from the mean of the measurements at 492 nm.

### 2.11. Molecular Analysis

To quantify CDV loads and cytokine responses at the molecular level, RT-qPCR analysis (38 slices derived from 4 dogs) was performed. RNA was isolated and purified from frozen PCLSs using the RNeasy^®^ Mini Kit (Qiagen, Hilden, Germany) as described [38]. Measurement of the optical density at 260 nm with a spectrophotometer (Multiskan™ GO microplate spectrophotometer, µDrop™ plate, SkanIt™ software version 5.0.0.42, Thermo Fisher Scientific, Braunschweig, Germany) allowed calculation of the obtained RNA amount and absorbance at 260 nm and 280 nm was used to assess the purity of RNA.

Total RNA was transcribed into complementary DNA (cDNA) using the Omniscript^®^ Reverse Transcription Kit (Qiagen), RNaseOUT™ Recombinant Ribonuclease Inhibitor (Invitrogen, Carlsbad, CA, USA), and random primers (Promega Corporation, Madison, WI, USA) following the manufacturer’s instructions as described [38].

Primer sequences and plasmids for generation of standard dilutions and qPCR products (Eurofins Genomics, Ebersberg, Germany) were taken from the literature and have been described in detail in a previous publication [38]. Standard dilutions were either generated from extracted PCR products or plasmids, and RT-qPCR analysis using the AriaMx Real-Time PCR System (Agilent Technologies; Agilent Aria software version 1.71) was performed as described [38].

### 2.12. Statistical Analysis

Statistical analysis was performed using the Kruskal–Wallis *H* test in the program SPSS for Windows^TM^ (version 28.0.1.1 (15), IBM^®^ SPSS^®^ Statistics, SPSS Inc., Chicago, IL, USA). *p*-values of ≤ 0.05 (*) were accepted as statistically significant. Correlation analysis for CDV and MHC-II was performed using Spearman’s rho correlation in SPSS. Graphs were created with GraphPad Prism (version 9.0.0. (121), GraphPad Software, San Diego, CA, USA) for Windows™.

## 3. Results

### 3.1. Morphology and Viral Loads

**Figure 2:**
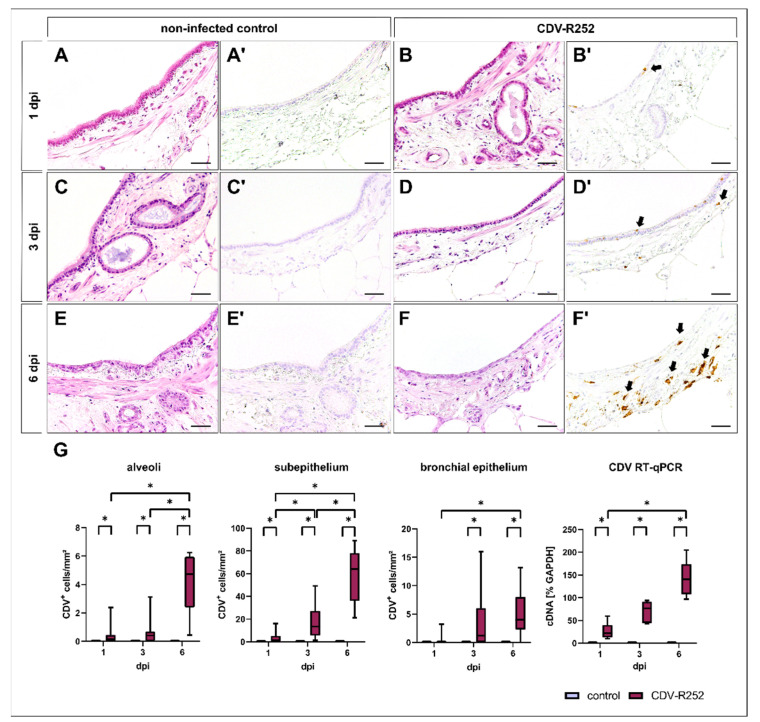
Morphology of canine precision-cut lung slices (PCLSs) and quantification of canine distemper virus (CDV) loads. Morphology remains stable during the cultivation period in noninfected (**A**,**C**,**E**) and CDV-infected (**B**,**D**,**F**) PCLSs, except from a mild attenuation of the bronchial epithelium and ciliary loss in both groups in the advanced cultivation period (**E**,**F**; HE stain). CDV antigen was not detected in control PCLSs (**A’**,**C’**,**E’**), while CDV-infected PCLSs showed increased viral loads during the infection period, mainly within the bronchial subepithelium (**B’**,**D’**,**F’**). Quantification of CDV antigen by immunohistochemistry and CDV cDNA by RT-qPCR revealed a progressive course of infection (**G**). Box and whisker plots show median and quartiles with maximum and minimum values. Significant changes are labelled by an asterisk (*p* ≤ 0.05, Kruskal–Wallis *H* test). dpi = days postinfection; GAPDH = glyceraldehyde 3-phosphate dehydrogenase; scale bars: 50 µm.

Morphology of PCLSs was assessed by light microscopy in HE-stained PCLS sections. PCLS tissue architecture was maintained during the infection period. With prolonged culture time, mild flattening of the bronchial epithelium with loss of cilia was observed in both groups (Figure 2A–F).

Viral loads in PCLSs at 1, 3, and 6 dpi were evaluated by immunohistochemistry. Staining revealed a progressive increase of CDV-infected cells in PCLSs over time in alveoli, bronchial subepithelial tissue (subepithelium), and the bronchial epithelium. CDV was predominantly located within the bronchial submucosa at all time points. Bronchial epithelial infection was rarely observed at 1 dpi but increased at 3 dpi (Figure 2A’–F’,G). Progressive CDV infection was confirmed by detection of viral RNA by two-step RT-qPCR (Figure 2G).

### 3.2. Cell Tropism and Ultrastructural Detection of Canine Distemper Virus

CDV was visualized in PCLSs by immunohistochemistry, in situ hybridization, and double immunofluorescence labelling, as well as by transmission electron microscopy.

**Figure 3:**
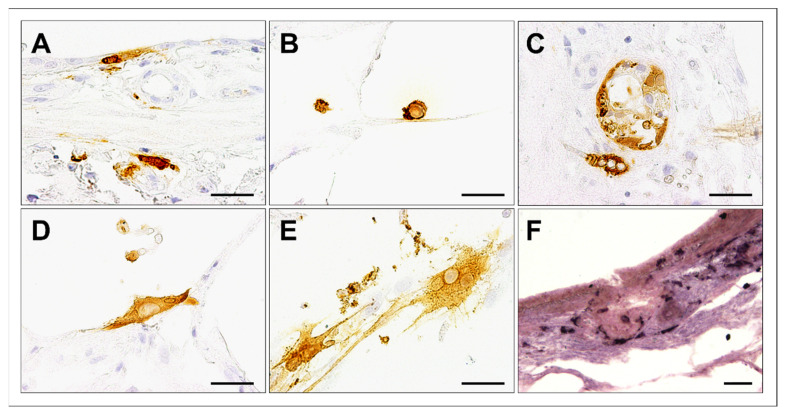
Cell tropism in canine distemper virus (CDV)-infected canine precision-cut lung slices (PCLSs) at day 6 postinfection. CDV antigen was detected in bronchial epithelial cells (**A**), alveolar macrophages (**B**), bronchial glands (**C**), and alveolar epithelial cells (**D**). Occasionally, multinucleated cells containing CDV antigen were observed (**E**). CDV RNA was detected by in situ hybridization (dark purple, **F**). Scale bars: 50 µm.

Immunohistochemistry and in situ hybridization showed positivity for CDV antigen and RNA in several cell types, including bronchial epithelial cells, alveolar macrophages, and bronchial glandular epithelial cells. Occasionally, CDV-infected PCLSs exhibited positive labelling of CDV antigen in single multinucleated cells at 6 dpi (Figure 3).

**Figure 4:**
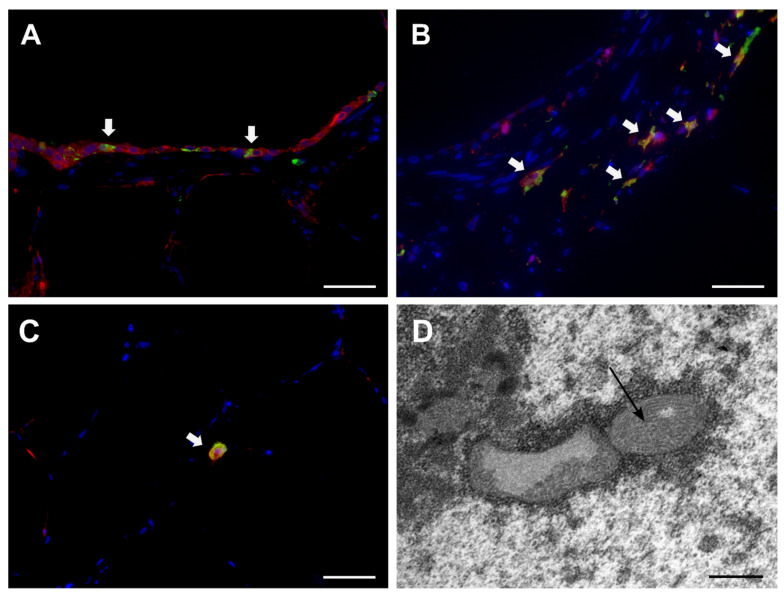
Detection of canine distemper virus (CDV) in PCLSs by immunofluorescence (**A**–**C**) and transmission electron microscopy (**D**). Immunofluorescence double-labelling detection of CDV antigen (green), cytokeratin (red, A), or Iba-1 (red, B) revealed CDV infection of epithelial cells (white arrows, **A**), peribronchial histiocytes (white arrows, **B**), and alveolar macrophages (white arrow, **C**) at day 6 postinfection. Nuclear counterstaining: bizbenzimide (blue). Scale bars: 50 µm. Intranuclear inclusions of CDV nucleoprotein filaments at day 3 postinfection (**D**, arrow). Scale bar: 500 nm.

Immunofluorescence double-labelling confirmed localization of CDV antigen within cytokeratin^+^ epithelial cells and Iba-1^+^ histiocytes (Figure 4 A–C). By transmission electron microscopy, CDV filaments were demonstrated in alveolar and airway epithelial cells as well as in leukocytes (Figure 4D).

### 3.3. Ciliary Activity and Viability

**Figure 5:**
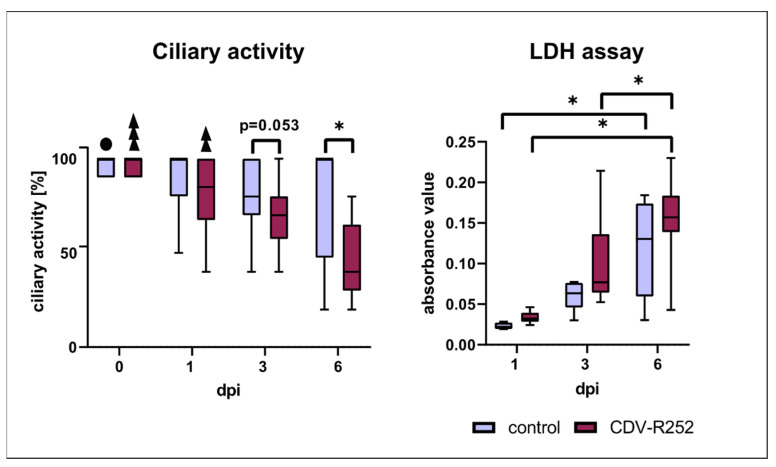
Ciliary activity and lactate dehydrogenase (LDH) assay. Box and whisker plots show median and quartiles with maximum and minimum values. Significant changes are labelled by an asterisk. Black sphere: significant difference to control at 3 dpi; three superimposed triangles: significant difference to CDV-infected samples at 1, 3, and 6 dpi; two superimposed triangles: significant difference to CDV-infected samples at 3 and 6 dpi (*p* ≤ 0.05, Kruskal–Wallis *H* test).

Ciliary beating activity, assessed by light microscopy prior to infection, was reduced at 3 dpi with a trend towards statistical significance (*p* = 0.053) and significantly lower in CDV-infected PCLSs compared to the uninfected control at day 6 postinfection, indicative of virus-mediated ciliary dysfunction. In both controls and the infected group, ciliary activity decreased significantly during the cultivation period. LDH activity within the supernatant, an indicator of cell damage, increased in both groups with prolonged infection time. However, no significant differences regarding cell viability were observed between controls and CDV-infected PCLSs (Figure 5).

### 3.4. Major Histocompatibility Complex Class II Expression

**Figure 6:**
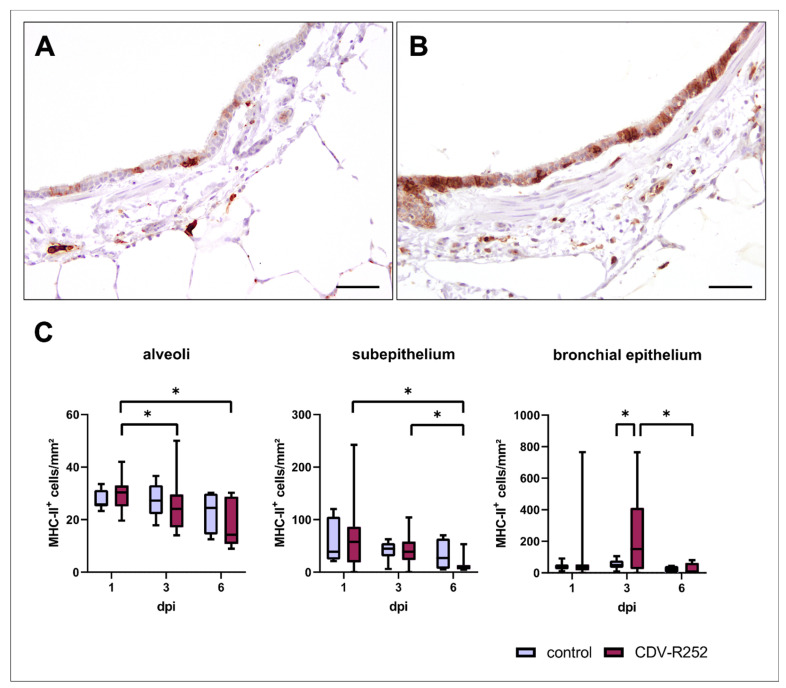
Spatiotemporal quantification of MHC-II antigen expression in precision-cut lung slices (PCLSs). Representative images of an uninfected (**A**) and canine distemper virus (CDV)-infected (**B**) PCLS at day 3 postinfection. An increased MHC-II expression was observed in the bronchial epithelium of CDV-infected PCLSs at day 3 postinfection when compared to controls (**C**). Box and whisker plots show median and quartiles with maximum and minimum values. Significant changes are labelled by an asterisk (*p* ≤ 0.05, Kruskal–Wallis *H* test). dpi = days postinfection. Scale bars: 50 µm.

MHC-II expression was evaluated by immunohistochemistry. CDV-infected PCLSs showed transiently increased MHC-II^+^ cell numbers within the bronchial epithelium at day 3 postinfection. Moreover, a significant decline of MHC-II expression was observed in the alveolar region of CDV-infected PCLSs at 3 and 6 dpi compared to 1 dpi, within the subepithelium at 6 dpi compared to 1 and 3 dpi, and within the bronchial epithelium at 6 dpi compared to 3 dpi (Figure 6). MHC-II expression correlated negatively with CDV loads within the bronchial subepithelium (correlation coefficient: −0.299) and positively within the bronchial epithelium (correlation coefficient: 0.357).

### 3.5. Cytokine Responses

**Figure 7:**
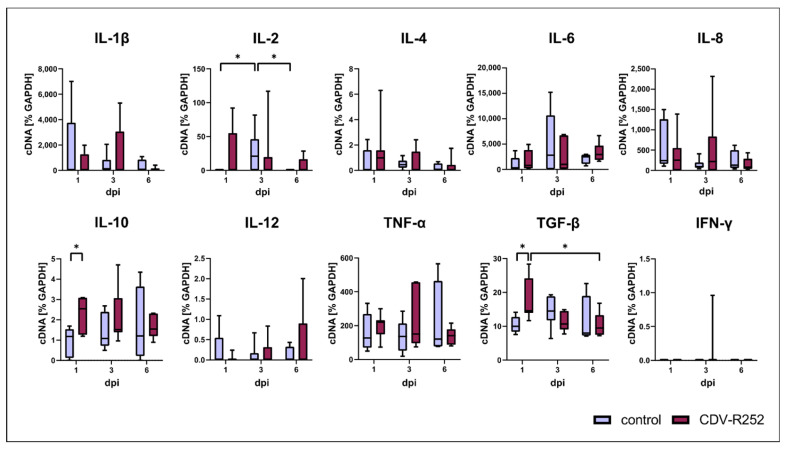
Cytokine expression in canine distemper virus (CDV)-infected precision-cut lung slices (PCLSs). IL = interleukin; TNF-α = tumor necrosis factor alpha; TGF-β = transforming growth factor beta; IFN-γ = interferon gamma. Significant changes are labelled by an asterisk (*p* ≤ 0.05, Kruskal–Wallis *H* test).

RT-qPCR data revealed a significantly increased transcription of the anti-inflammatory cytokines interleukin (IL)-10 and transforming growth factor beta (TGF-β) on day 1 postinfection. IL-2 mRNA levels were significantly increased in controls at day 3, followed by a decrease at day 6. However, no differences in IL-2 transcription were found between uninfected and CDV-infected PCLSs. No significant changes between CDV-infected and uninfected PCLSs were detected regarding IL-1β, IL-4, IL-6, IL-8, IL-12, tumor necrosis factor alpha (TNF-α), and interferon (IFN)-γ transcription (Figure 7). Data indicate that CDV elicits an inhibitory cytokine response in the respiratory tract upon infection.

## 4. Discussion

Despite the important role of the respiratory tract in morbillivirus diseases, the pathogenesis and immunopathology in the lung during CDV infection in dogs are poorly understood. In the present study, canine precision-cut lung slices were infected with CDV, demonstrating that infection influences local pulmonary defense responses by ciliary dysfunction and expression of the inhibitory cytokines IL-10 and TGF-β.

Data revealed a progressive CDV-infection by temporospatial analysis of viral loads and virus distribution in lung tissue ex vivo. CDV antigen was detected in both leukocytes and epithelial cells, thus resembling the cell tropism found in naturally infected dogs in vivo [38,43]. Moreover, CDV nucleocapsid was demonstrated by transmission electron microscopy in canine CDV-infected PCLSs. Interestingly, most CDV-infected cells were located within the airway subepithelium expressing the histiocytic marker Iba-1, indicative of myeloid cell infection. Previous CDV infection studies using canine lung explant cultures identified SLAM^+^ immune cells as the main target cells of the virus [32,33]. Similarly, a preferential infection of lymphoid cells was observed in MeV-infected lung slices of macaques, mainly within the bronchus-associated lymphoid tissue [32]. In CDV-infected canine air–liquid interface (ALI) cultures, epithelial infection was enhanced by cocultured CDV-infected SLAM^+^ histiocytic cells (DH82 cells) on the basolateral side of the epithelium [28]. These results are in accordance with the current hypothesis of morbillivirus pathogenesis in the lung, which suggests that SLAM-expressing immune cells are infected prior to epithelial cells [44,45,46]. Possible explanations for preferential infection of SLAM^+^ cells are a better accessibility and affinity of the SLAM receptor compared to nectin-4, which is hidden in the tight junctions of the bronchial epithelium [47]. Additionally, an upregulation of SLAM expression in immune cells has been demonstrated upon CDV infection of dogs and MeV-infection of macaques, further facilitating viral entry [48,49]. Recently, low-density lipoprotein receptor-related protein 6 (LRP6) has been identified in vitro as an entry receptor for the attenuated CDV strain Onderstepoort [50]. Its role in pulmonary CDV infection remains to be elucidated.

Propelling pathogens from the epithelial surface towards the outer environment by ciliary movement is one important defense mechanism of the respiratory innate immune system. Several viruses, such as SARS-CoV-2 or respiratory syncytial virus (RSV), have been shown to impede this mucociliary escalator, facilitating their entry into the host [51,52]. Similarly, a decrease of ciliary beating activity was observed in CDV-infected PCLSs when compared to uninfected controls in the present study. Due to similar morphology and viability of controls and CDV-infected PCLSs, it is speculated that a functional impairment, rather than a numerical decrease of ciliated cells, contributes to this finding. Inhibition of ciliary beating without changes in tissue integrity was also observed in ex vivo airway cell cultures infected with RSV, starting at 4 dpi [53]. Another study showed ciliary dyskinesia with an unaffected ciliary beating frequency following RSV-infection in human nasal epithelial ALI cultures [54]. Reduced ciliary activity (ciliostasis) matches with previous observations in transcriptome analysis of acutely CDV-infected lung tissue of dogs, showing a downregulation of genes involved in ciliary and epithelial function, such as dynein axonemal intermediate chain 3, HOATZ cilia, and flagella-associated protein [38]. However, additional mechanisms may be involved in ciliary dysfunction in vivo, as acute CDV infection manifests as necrotizing bronchiolitis in association with proinflammatory immune responses, and oxidative stress caused by infiltrating leukocytes [38,55,56].

Antigen presentation to naïve CD4^+^ T cells by MHC-II is a major factor in the recruitment and activation of adaptive immune responses [57]. MHC-II expression was increased in the bronchial epithelium of CDV-infected PCLSs at day 3 postinfection. Similarly, a transient upregulation of MHC-II during the acute and subacute infection phase, followed by a decrease at later disease phases, was found in lung tissues of naturally CDV-infected dogs [38]. Increased numbers of MHC-II^+^ cells were also reported in the lungs of CeMV-infected dolphins [58]. MHC-II upregulation might be an advantage in combating infection in vivo, but its effect in PCLSs remains to be questioned, as viral loads increased at 6 dpi and MHC-II-responding immune cells cannot infiltrate. The decreased MHC-II positivity in CDV-infected PCLSs on day 6 postinfection might be due to reduced viability of the cells. However, modulation of antigen-presenting capacity with reduced MHC-II production has been described in vitro in CDV-infected dendritic cells, which is proposed as an evasion strategy from adaptive host immune responses [59]. Therefore, since viral loads increased in the course of infection, a directly virus-induced inhibitory effect on MHC-II expression should be considered. In contrast to the PCLS model, which lacks supply by infiltrating blood-derived immune cells, decreased MHC-II expression in advanced pulmonary lesions in dogs are supposed to be attributed to disease remission, viral clearance, and reduced production of proinflammatory cytokines [60].

Cytokines orchestrate and modulate antiviral immune responses, significantly influencing the outcome of viral infection and inflammatory changes. RT-qPCR analysis showed an increase of IL-10 and TGF-β transcription in CDV-infected PCLSs at day 1 post infection. IL-10 and TGF-β act as immunomodulatory and anti-inflammatory cytokines. They influence cytokine expression and cell surface receptors on myeloid cells and inhibit T helper 1 (Th1) cell responses, thus regulating inflammatory responses and tissue homeostasis [61]. In particular, IL-10 is a potent inhibitory cytokine and antagonizes proinflammatory cytokines. Therefore, it limits and terminates inflammation-induced tissue damage and promotes disease remission, correlating with a T helper 2 (Th2) lymphocyte response and augmented M2-type macrophage polarization. Additionally, it modulates antigen-presenting capacities by inhibition of MHC-II antigen expression [61,62,63]. Suppressing the maturation of dendritic cells and Th1 cell responses, IL-10 contributes to an impairment of viral clearance mechanisms and leads to an anergic state of lymphocytes and subsequent viral persistence [64,65]. IL-10 is secreted by monocytes, macrophages, dendritic cells, B cells, various T cell subsets, and epithelial cells [66,67,68,69]. It may function in an autocrine manner, e.g., on dendritic cells and macrophages, which is associated with subsequent inhibition of downstream immune responses and polarization of macrophages towards an anti-inflammatory M2 phenotype [70,71]. Impaired T cell responses due to IL-10 secretion have been demonstrated in the context of various viral diseases, e.g., lymphocytic choriomeningitis, acquired immunodeficiency syndrome, hepatitis C, and Theiler’s murine encephalomyelitis [72,73,74,75,76]. TGF-β shows a similar inhibitory function as IL-10, although its effect is not as strong [20]. In the lung, TGF-β is involved in wound healing, fibrosis, and differentiation of naïve CD4^+^ cells into Th17 cells and regulatory T cells following tissue injury [77]. It can also enhance viral replication by induction of cell cycle arrest, as demonstrated in RSV infection [78]. The role of IL-10 and TGF-β during CDV infection is complex and not yet fully elucidated. In agreement with the present findings, in vitro studies using canine peripheral blood mononuclear cells revealed increased IL-10 levels at day 1 after exposure to CDV [79]. In CDV-infected dogs, increased levels of IL-10 were found in the cerebrospinal fluid, and increased TGF-β levels in blood samples [80,81]. Moreover, in vitro CDV infection of dendritic cells revealed an increased IL-10 transcription. The concomitant downregulation of antigen-presenting capacities implicates an IL-10-associated impairment of adaptive immune responses, potentially favoring viral persistence [59]. Likewise, in measles patients, increased IL-10 levels were observed in blood samples in the acute stage of disease [82,83,84]. Interestingly, the present findings contrast with results in lung tissue of naturally CDV-infected dogs with manifest pulmonary disease, which might be due to a lack of signals by extrapulmonary leukocytes in PCLSs. During natural CDV infection, a proinflammatory cytokine profile with increased TNF-α, IL-6, and IL-12 levels can be observed in canine lung tissues, which is primarily evoked by infiltrating immune cells [38]. Thus, the PCLS model mimics the early initial phase of CDV infection in resident lung cells before the recruitment of leukocytes, explaining the different cytokine profiles found ex vivo and in lungs of CDV-infected dogs. The function of IL-10 and TGF-β as anti-inflammatory cytokines and their contributory role to impairing antiviral immunity renders these molecules a potential target for antiviral therapy during initial CDV infection [59].

PCLSs offer the advantage to study early local immune responses in resident cells of pulmonary tissue. Nonetheless, the present model possesses some limitations due to variations likely attributed to differences in breed, sex, and age of the studied dogs, which were not raised under gnotobiotic laboratory conditions. However, the increased use of ex vivo platforms such as PCLSs more closely mimics natural conditions to investigate infectious diseases, and provides a promising answer towards rising demands of animal experiment reduction according to the 3R principles and, depending on the question investigated, offers a scientifically sound complementation or alternative to animal experiments.

In conclusion, progressive CDV infection has been demonstrated for the first time in canine PCLSs, an ex vivo model of local pulmonary defense responses during viral infection. The expression of anti-inflammatory and inhibitory cytokines within the lung may enhance viral replication during the initial phase of canine distemper. Moreover, CDV infection negatively influences ciliary function. Obtaining further insights into virus–host interactions in the lung is essential for a deeper understanding of pathogenesis and the development of novel treatment strategies in morbillivirus-induced disease.

## Figures and Tables

**Table 1:** Primary antibodies used for immunohistochemistry and immunofluorescence.

Epitope	Source	Cat. No.	Species	Clone	Dilution
CDV-N	Santa Cruz Biotechnology, Dallas, TX, USA	sc-57660	Mouse	DV2-12	1:10,000 * 1:100 **
MHC-II	Dako, Glostrup, Denmark	M0746	Mouse	TAL.1B5	1:80 *
CK	Dako, Glostrup, Denmark	Z0622	Rabbit	Polyclonal	1:50 **
Iba-1	Invitrogen™, Thermo Fisher Scientific, Langenselbold, Germany	PA5-27436	Rabbit	Polyclonal	1:200 **

Cat. No. = catalogue number; CDV-N = canine distemper virus nucleoprotein; MHC-II = major histocompatibility complex class II; CK = cytokeratin; Iba-1 = ionized calcium-binding adapter molecule 1; * dilution used for immunohistochemistry; ** dilution used for immunofluorescence.

## Data Availability

All relevant data are included in the manuscript or can be obtained from the authors on reasonable request.

## Supplementary Materials

The following supporting information can be downloaded at: https://www.mdpi.com/article/10.3390/v15040834/s1, Table S1: Details of dogs used for precision-cut lung slices (PCLSs); Figure S1: Scheme of evaluation of ciliary beating activity by light microscopy; Video S1: Exemplary video of ciliary beating in PCLS.
