# Supplementary material for: Canine Distemper Virus Alters Defense Responses in an Ex Vivo Model of Pulmonary Infection
Source: Viruses. 2023 Mar 24;15(4):834. doi: 10.3390/v15040834 (PMC10144441; doi:10.3390/v15040834)
Supplement: Supplementary file 1 [file viruses-15-00834-s001.zip › Supplementary_Material.pdf]

Supplementary Material

Canine Distemper Virus Alters Defense Responses in an *Ex Vivo* Model of Pulmonary Infection

Elisa Chludzinski [1,3], Małgorzata Ciurkiewicz [1], Melanie Stoff [1], Johanna Klemens [1], Johannes Krüger [1], Dai-Lun Shin[2], Georg Herrler [2,*], Andreas Beineke [1,3,*,]**

[1] Department of Pathology, University of Veterinary Medicine Hannover, 30559 Hannover, Germany
[2] Institute of Virology, University of Veterinary Medicine Hannover, 30559 Hannover, Germany
[3] Center for Systems Neuroscience (ZSN), 30559 Hannover, Germany

* Both authors have contributed equally last (G.H., A.B.)
** Correspondence: andreas.beineke@tiho-hannover.de (A.B.)

Supplementary Material:

This file includes: Supplementary Table S1, Supplementary Figure S1

Supplementary Table S1: Details of dogs used for precision-cut lung slices (PCLSs)

Dog No	Age (years)	Sex	Breed
1	13	m	Petit Basset Griffon Vendeen
2	6.5	mc	Labrador
3	18	m	mixed breed
4	8	f	mixed breed
5	12	fs	mixed breed
6	11	fs	Australian Shepherd

m = male; mc = male castrated; f = female; fs = female spayed

Supplementary Figure S1:

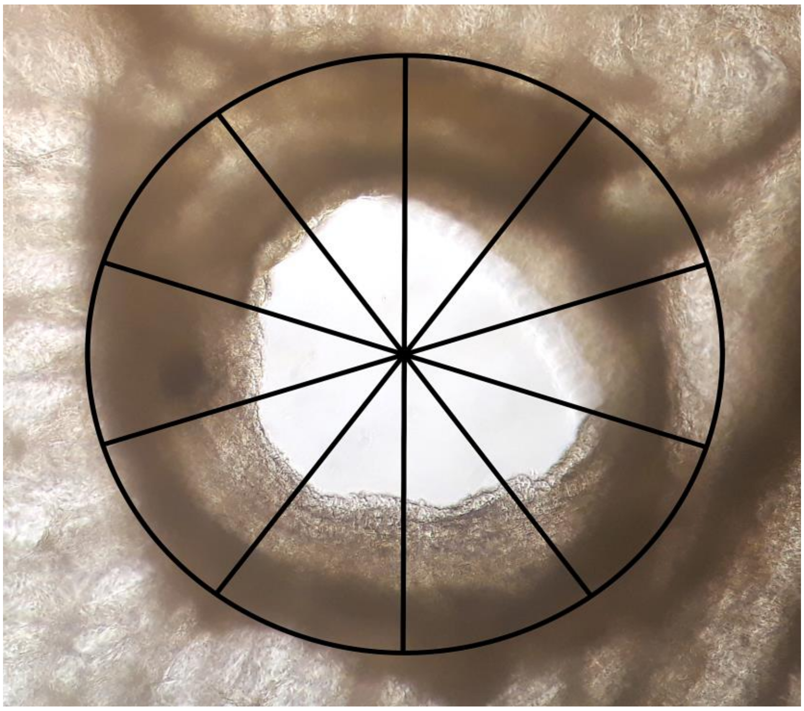

Supplementary Figure S1: Scheme for the evaluation of ciliary beating activity by light microscopy. To evaluate the percentage of ciliary beating activity, the bronchus is divided into ten equally-sized segments.
